# Coping strategies and personal growth: The case of Palestinian refugees in Shatila camp, Lebanon

**DOI:** 10.3389/fpsyg.2023.1083998

**Published:** 2023-03-09

**Authors:** Sara Al Beainy, Karma El Hassan

**Affiliations:** Department of Education, American University of Beirut, Beirut, Lebanon

**Keywords:** stressors, coping strategies, emotion-based coping, problem-based coping, avoidant coping, growth

## Abstract

This study investigated the relationship between coping strategies used by adolescent refugees in the Palestinian refugees’ Shatila camp in Lebanon and posttraumatic growth. Moreover, the study explored and predicted the impact of coping strategies utilized by adolescent Palestinians in Shatila camp, Lebanon on their personal growth and psychological well-being. Data were collected using two questionnaires and a checklist: (a) LEC-5 checklist as an assessment tool to make sure that all the participants have faced or experienced stressful events, (b) questionnaires including the Ways of Coping Questionnaire (WCQ) to find out the style of coping refugees used, and (c) Posttraumatic Growth Inventory (PTGI) to identify the factors of growth refugees developed as a result of using different coping strategies. Sixty adolescent refugees at one of the centers in the camp (31 females and 29 males) who benefited from counseling services participated in the study. Adolescent refugees’ performance on the checklist and questionnaires revealed the prevalence of stressors among the refugees. The coping strategies mostly utilized were problem-focused coping strategies, as there was a correlation between its factors and some coping strategies, and there were coping strategies used that predict the development of growth among. Finally, as for the counseling and training programs and services, interventions and guidance services seem to better prepare refugees to handle and cope with the stress that they encounter to develop personal growth.

## Introduction

Psychological well-being reflects humans’ positive mental state, such as satisfaction or happiness. According to Diener (2000), there are two crucial facets of psychological well-being. The first of these facets refers to the extent to which people experience positive emotions and feelings of happiness, and the second refers to the extent people adapt to influences that affect their cognitive evaluation of their lives. Six factors reflect psychological well-being: ability to manage complex environments, self-acceptance, sense of autonomy, continued personal growth, the pursuit of meaningful goals, and establishment of quality ties to others.

According to the World Population review, more than one million refugees and asylum seekers been hosted in Lebanon from different nationalities: Palestinian, Syrian, Iraqi, and others. UNICEF (2022) reported that there were more than three million Syrian refugees in Lebanon escaping violence in their own country. As a result, the conditions of the camps in Lebanon, as well as the Shatila camp became extremely bad with overcrowded shelters (Al-Hroub, 2014; Sayegh, 2015).

Since the establishment of Shatila in 1949, the camp has been one of the sites that encompasses and confines a large number of refugees. In the beginning, during the early years of its founding, Shatila enclosed around 500 residential units, but this picture failed to remain as such for a protracted time. The camp has immensely grown since its establishment. There were 10,849 UNRWA registered refugees in the Shatila camp, as of June 2018. Yet, this number does not represent the definite number of Palestinian refugees present in the camp since some of them may have left over the years (Sayegh, 2015).

UNRWA provides refugees with financial support and assistance (Al-Hroub, 2011, 2015; Sayegh, 2015). Since 2011, when the Syrian crisis began, many Syrians settled in refugee camps in Lebanon (Al-Hroub et al., 2020; Al-Hroub, 2022). As a result of this shift, UNRWA underpaid Palestinian refugees, creating gaps in their assistance (Steflova, 2017). Camp dwellers found it difficult to locate sustainable jobs.

Research has shown that stressful events ranging from wars and combat actions, natural disasters, illness, and dramatic life changes affect the psychological well-being of an individual causing stress, anxiety, and depression (Chan et al., 2016). According to Boals and Schuler (2019), refugees experience stressful events, mainly war, which affect their psychological well-being. Moreover, stressful experiences affect a person’s ability to function, as it affects their existence in the long term. On the other hand, some studies show that individuals can overcome stress through their coping strategies, which leads them to posttraumatic growth (Boals and Schuler, 2018). Individuals differ in their interpretations of the stressful event, and therefore their reactions and coping strategies are also likely to be different (Khamis, 2012). Several studies indicated that personal variables such as age and gender also influence how individuals appraise, react to, and cope with stressful situations (Khamis, 2015). Based on our search, we were not able to find any study that examined the situation of Palestinian refugees in Lebanon with relevance to coping strategies and how they were able to achieve their growth after facing stressful factors (PTG).

Some research tackles the issue of stressors that refugees face (Khamis, 2012, 2015); other research tackles the posttraumatic growth of refugees or their well-being after experiencing stress (Rizkalla and Segal, 2018). Research shows the relation between refugees’ well-being and/or the stressors they face and their academic performance. Moreover, the research examines the posttraumatic growth of refugees, where they deal with stressors, overcome them, and succeed in their lives. However, the posttraumatic growth and coping strategies of Palestinian refugees were not studied in Lebanon.

The literature demonstrates the effects of coping strategies on youths’ mental health and well-being, and it has revealed that active coping strategies are associated with better mental health and well-being, whereas the use of avoidant coping is related to various undesirable outcomes (Runtz and Schallow, 1997; Tremblay et al., 1999; Kraaij et al., 2003; Flett et al., 2012). This study targets the adolescent age group, as it is a critical stage where individuals during this period vary in identifying their stressors and the way they cope with their stress. Adolescents use a variety of coping strategies in order to reduce or manage the physical, psychological, or social harm of stressors (Arslan, 2017). There has been recent attention to adolescents utilizing coping strategies (Williams and McGillicuddy-De Lisi, 1999). Research findings indicate that youths’ mental health and psychological well-being are affected by their coping strategies, and better mental health is due to active coping strategies. On the other hand, the use of avoidant coping is related to various undesirable outcomes (Runtz and Schallow, 1997; Tremblay et al., 1999; Kraaij et al., 2003; Flett et al., 2012). In addition, the use of distinct dimensions of coping strategies affects mental health differently. For example, problem-focused coping strategies are considered a significant component of resilience in the face of negative life events (Carroll, 2013); thus, this coping style protects youths’ mental health and well-being. On the other hand, avoidant coping makes things worse; it affects youths’ mental health negatively and may result in various mental health problems (Arslan, 2017).

According to Arslan (2017), harmful experiences can seriously affect the development of an individual’s coping strategies; however, it is not obvious how different coping strategies are related to individuals’ growth and psychological well-being. In Shatila, studies are usually conducted by international non-governmental organizations (NGOs) to assess poverty and basic human rights. Teachers, parents, social workers, non-governmental organizations, and even center directors can have a significant impact in assisting these affected individuals by considering their unique differences and their ability to cope with and face stressors or traumatic events. Counselors can show and teach adolescents how to prevent negative feelings that might affect and disrupt their well-being (Patros, 1989). Moreover, refugees would be motivated and supported to choose the coping strategies that would guide their way to growth and psychological well-being as they will be able to understand and identify the different coping strategies. In addition, they will be encouraged to find out what coping dimension suits their situations and what coping strategies they were already using when facing stress to maintain healthy well-being to develop personal growth.

Mental health specialists are interested in counseling because it prevents problems from worsening and lessens the effects of emotional disturbance and academic failure (Henderson and Thompson, 2015). Literature shows that counselors’ practice improves when they know and address clients’ needs (Astramovich et al., 2013). Thus, completing this study adds to characterizing the coping mechanisms employed by Palestinian teenage refugees in the Shatila camp and the relationship between these coping strategies and the posttraumatic growth of these refugees.

## The current study

This research study investigated the type of coping strategies whether emotional, problem-focused, or avoidant utilized by adolescent Palestinian refugees who have experienced stressful events. It also attempted to find out if using coping strategies results in personal growth: posttraumatic growth in particular. More specifically the findings of this research were guided by the following questions: (a) What are the coping strategies used by adolescent Palestinian refugees in Shatila camp, Lebanon?, (b) What is the relationship between the coping strategies used and the adolescent Palestinian refugees’ posttraumatic growth?; and (c) What are the predictors of posttraumatic growth among adolescent Palestinian refugees in Shatila camp?

## Methods

### Participant recruitment

In this study, there were sixty randomly selected adolescents aged 15 to 17 from the population, at the Shatila refugees’ camp, who enrolled or were enrolled in one of the Shatila centers. The center was founded in 1997 by a long-time Shatila resident, and it aimed at developing children’s and youths’ potentials and providing them space and opportunity to learn and engage in activities; their main goal has been protecting the child’s right to learn and play in safety. The Center still works under the International Child and Human Rights Convention in cooperation with the United Nations International Children’s Emergency Fund (UNICEF), Save the Children (Sweden), United Nations Educational, Scientific and Cultural Organization (UNESCO), and other child protectors. The Center welcomes refugees in its two sites at Shatila and Nahr el-Bared refugee camps; it is a non-governmental organization and caters to all nationalities residing within the camp. The adolescents who participated in the study should have attended counseling sessions or practices or requested guidance from members of the Center, and not only engaged in educational classes. Thirty-one participants were identified as females and twenty-nine as males. There were seventeen adolescents of age 15, twenty-four adolescents of age 16, and nineteen participants aged 17. Thirty-five of the participants in the study were current accessors of the center’s services, while twenty-five adolescent participants were past accessors to the Center’s counseling programs.

### Procedures

To maintain the reliability of the study, the Principal Investigator (PI) had training and was certified by “The Collaborative Institutional Training Initiative” before conducting the study. The researcher completed courses in “Social and Behavioral Research” and “Social and Behavioral Responsible Conduct of Research.” The PI submitted the proposal to the Institutional Research Board (IRB) at American University of Beirut for approval. Upon receiving IRB approval, the PI visited the Center several times and worked according to the Center’s code of conduct. First, the Center in Shatila was contacted through a formal letter to obtain permission to conduct the study. The pilot study was conducted; then the questionnaires were completed by a random sample of selected participants. Following the IRB guidelines, the package was handed to participants; it included the three data collection tools that were be administered in Arabic (mentioned in the Instruments section).

To avoid any perception of undue influence and follow the IRB guidelines, the PI asked the director of the Center to help in assigning the secretary or the director’s assistant to contact the parents to seek their agreement. As a result, they approved sharing their contact information with the research team. The parents were invited to the Center; they were handed parental permission included in the participation package for their adolescents. Transportation was also provided for parents to and from the Center when needed. They were sitting in groups of five to six in a big room; covid precautions and safeguards, as advised by the World Health Organization (WHO) such as social distancing, were implemented when explaining to them the purpose of the study and seeking their permission. After that, the adolescents whose parents agreed to their participation were asked by visit the Center to the secretary to visit the Center the day after. The adolescents were both past and current accessors to the Center’s services. Past applicants who were unable to reach the Center were provided with transportation. Participants were grouped in a large room with social distancing to maintain all safety measures. The researcher was present and read special instructions which were administered same to all participants. The researcher guided the participants and explained to them the purpose of the study and sought their approval. This was done during all the visits to the Center.

### Instruments

Life Event Checklist-5 (LEC-5), Ways of Coping Questionnaire (WCQ), and Posttraumatic Growth Inventory (PTGI) are originally English scales that were administered in this study for data collection purposes. Data were collected using a modified version of the LEC-5. An item that states other sources of stress was added to the LEC-5. Based on the guidelines of the International Test Commission (ITC) (2016), back-and-forth translation and adaptation processes were used. Experienced translators translated the three tools backward to Arabic; then, another group of translators back translated them to English. Items of the translated tools were compared to the original English tools. To validate the checklist, the WCQ, and the PTGI, two bilingual experts and educational psychologists participated in modifying and adapting the tools to the context of the study. The adaptation was a very important procedure designed to examine whether all items were clear, understandable, and valid with regard to Palestinian refugees in Shatila camp. It was intended to have these scales translated into Arabic and adapted to suit Shatila camp adolescents as Arabic is their mother tongue.

#### Life Event Checklist-5

A large body of research supports the reliability and construct validity of the LEC-5. According to the National Center of PTSD, LEC demonstrated convergent validity when measuring different levels of exposure to potentially traumatic events. The checklist evaluated the respondent’s exposure to a wide range of stressful and traumatic experiences (Gray et al., 2004). The LEC-5 used in this study was composed of 17 items; 16 of the events are known to potentially result in distress or Post Traumatic Stress Disorder (PTSD). The last item was added to adjust to the situation of the campsite, and it assessed any other extraordinary stressful event not mentioned in the first 16 items. Participants responded to stressful events by checking any of the possible choices. The scale ranges from happened to me, witnessed it, learned about it, not sure, and does not apply. The reliability of LEC-5 in this study was measured 0.71 using Cronbach’s alpha.

#### Ways of Coping Questionnaire

The Ways of Coping Questionnaire (WCQ) designed by Folkman and Lazarus (University of California, 1985) was developed from the Ways of Coping Checklist (WCC) by deleting and rewording several items as well as changing its format to a four-point Likert-type scale. The WCQ is a 66-item Likert scale questionnaire. The items were distributed in a random way reflecting the coping strategies that were used by individuals. This instrument, the WCQ reflects the coping processes that people utilize when a stressful encounter happens. Participants rate to what extent they used the process to cope with their problems. The scale ranges from 0 (not used), 1 (used somewhat), 2 (used quite a bit), to 3 (used a great deal). Five out of eight subscales measure problem-focused coping strategies. They are (1) Planful problem-solving, (2) Seeking social support, (3) Confrontation, (4) Accepting responsibility, and (5) Positive reappraisal. However, the other three subscales measure emotion-focused coping strategies. They are the following: (1) Distancing, (2) Escape/avoidance, and (3) Self-controlling. Each subscale is scored by averaging the questions of 4-point Likert scale responses for each of the subscale items. A large body of research supports the reliability of the WCQ (e.g., Folkman et al., 1986). The reliability of WCQ in this study was 0.67 using Cronbach’s alpha.

#### Posttraumatic Growth Inventory

Posttraumatic Growth Inventory (PTGI) was utilized as it measures the growth of a person after being exposed to trauma or any other stressful event. PGI theory explains the aspect of personal growth that is specifically both intentional and conscious; as it is the promising antecedent of well-being and optimal functioning (Robitschek, 1998; Robitschek et al., 2012; as cited by Weigold et al., 2013). The reliability of the whole PTGI scores using Cronbach’s alpha was 0.89 (Rizkalla and Segal, 2018). According to Tedeschi and Calhoun (2004), posttraumatic growth requires effortful cognitive processing. Participants must identify or rate the areas of growth they have witnessed within themselves after they have lived through a crisis or a stressful event. The PTGI scores are distributed on different factors, and each factor resembles a subscale. There are five factors of PTG. The factors are identified as the following: (1) relating to others, (2) new possibilities, (3) personal strength, (4) spiritual change, and (5) appreciation of life (Boals and Schuler, 2018). Each of the items of the PTG questionnaire reflects one of the factors. The PTGI has demonstrated construct validity in many studies of refugee populations (Kroo and Nagy, 2011; Chan et al., 2016; Sleijpen et al., 2016) and traumatized populations from the center East (Kira et al., 2012; Davey et al., 2015; Rizkalla et al., 2017; as cited by Rizkalla and Segal, 2018). The reliability of PTGI in this study was 0.92 using Cronbach’s alpha.

### Data analysis

Correlational analyses were performed to examine the relationship between coping strategies and the posttraumatic growth of Palestinian adolescent refugees. Moreover, regression analysis was done against various coping strategies to identify the variables/predictors that account for the largest variance in the Posttraumatic Growth of adolescent refugees in the Center, Shatila.

## Results

### Life stressful events

Results of respondents’ answers to the Life Event Checklist-5 Scale revealed that all the 60 adolescent participants reported that they have lived a stressful event whether they experienced or witnessed it. Table 1 presents the frequency of stressful events experienced by Palestinian participant refugees in the Shatila camp. The most frequent stressful event was the pandemic (*F* = 52) followed by Fire or Explosion (*F* = 42). Sudden accidental death was marked as one of the frequent stressing factors among refugees (*F* = 33). Serious accidents at work, at home, or during recreational activity were also cited as a frequent stressing factor among refugees (*F* = 29). Palestinian refugees also reported that “other stressing events or experiences” was a stressing factor witnessed by them (*F* = 27).

**Table 1:** Frequency of life stressful events among Palestinian refugees in Shatila camp.

Life stressful events	Happened to me	Witnessed it	Learned about it	Part of my job	Not sure	Does not apply
1. Natural disaster (e.g., flood, hurricane, tornado, earthquake)	_	5	19	1	12	23
2. Fire or explosion	42	8	4	1	4	1
3. Transportation accident (e.g., car accident, boat accident, train wreck, plane crash)	18	9	7	1	14	11
4. Serious accident at work, home, or during recreational activity	29	5	4	1	13	8
5. Exposure to toxic substances (e.g., dangerous chemicals, radiation)	11	1	8	_	13	27
6. Physical assault (e.g., being attacked, hit, slapped, kicked, or beaten up)	15	8	3	_	3	31
7. Assault with a weapon (e.g., being shot, stabbed, or threatened with a knife, gun, or bomb)	9	9	2	_	4	36
8. Combat or exposure to a warzone (in the military or as a civilian)	14	9	3	1	7	26
9. Captivity (e.g., being kidnapped, abducted, held hostage, or prisoner of war)	6	6	5	_	8	35
10. Life-threatening illness or injury	12	10	3	_	11	24
11. Severe human suffering	4	4	4	1	6	41
12. Sudden violent death (e.g., homicide, suicide)	6	12	6	1	10	25
13. Sudden accidental death	33	4	2	1	6	14
14. Serious injury, harm, or death you caused to someone else	10	2	3	_	11	34
15. Pandemic. COVID-19	52	1	1	_	3	3
16. Any other very stressful event or experience	_	27	1	_	11	21

### Ways of coping among refugees

Each of the items in the questionnaire represented a coping strategy that adolescent refugees utilized. The items of the Ways of Coping Questionnaire were distributed in 8 scales: (1) *Confrontive coping*, (2) *Distancing*, (3) *Self-controlling*, (4) *Seeking social support*, (5) *Accepting responsibility*, (6) *Escape-Avoidance*, (7) *Planful problem-solving*, and (8) *Positive reappraisal*. Descriptive statistics were used to report the most prevalent coping strategy among Palestinian adolescent refugees. Table 2 reports the percentages of various coping strategies utilized by the participants. All the coping strategies were being *used quite a bit*. The participants’ responses *used a great deal of* reported low percentages. The highest percentages of coping strategies among Palestinian adolescent refugees were those of Positive reappraisal and Planful problem-solving. Seventy-eight percent (78%) of the adolescents reported using positive reappraisal and seventy-five percent (75%) of the adolescent participants reported using Planful problem-solving to face their stressful events. Subsequently, sixty percent (60%) of these adolescents also reported seeking social support while coping with their hardships.

**Table 2:** Coping strategies among adolescent participants (*N* = 60).

Coping strategy	Percentage			
	0	1	2	3
Confrontive coping	3.4%	45%	44.9%	6.7%
Distancing	8.4%	35%	51.6%	5.1%
Self-controlling	0%	26%	74%	0%
Seeking social support	1.7%	35%	60%	3.3%
Accepting responsibility	0%	28.4%	68.4%	3.3%
Escape-avoidance	6.7%	46%	38.3%	0%
Planful problem-solving	0%	18.2%	75.1%	6.7%
Positive reappraisal	1.7%	15.1%	78.3%	5%

The scale ranges from 0 (not used), 1 (used somewhat), 2 (used quite a bit), to 3 (used a great deal).

### Posttraumatic growth among refugees

The results showed that several posttraumatic growth factors were prevalent among Palestinian adolescent refugees, and the percentages showed that all the posttraumatic growth factors were experienced and developed to a moderate degree among the participants. Table 3 reports the percentages of posttraumatic growth factors among the participants. Relating to Others PTG factor (63.3%) and Personal growth PTG factor (63.3%) recorded the highest scores among Palestinian adolescents, Appreciation of life recorded (50%), and New Possibilities recorded (48.4%). The lowest percentage was for Spiritual change (35%).

**Table 3:** Posttraumatic growth of life stressful events among Palestinian refugees in Shatila camp (*N* = 60).

Posttraumatic growth factors	Percentage					
	0	1	2	3	4	5
Relating to others	5%	34.9%	11.7%	63.3%	0%	0%
New possibilities	1.7%	21.6%	13.4%	48.4%	0%	0%
Personal growth	0%	5%	31.7%	63.3%	0%	0%
Spiritual change	6.7%	46.7%	11.6%	35%	0%	0%
Appreciation of life	26.6%	23.4%	0%	50%	0%	0%

0 (I did not experience this change), 1 (I experienced this change to a very small degree), 2 (I experienced this change to a small degree), 3 (I experienced this change to a moderate degree), 4 (I experienced this change to a great deal), 5 (I experienced this change to a very great degree).

### Correlation between coping strategies and posttraumatic growth

Correlation analysis was used to investigate the relationship between coping strategies and posttraumatic growth. The results yielded significant correlations between all coping strategies and posttraumatic growth factors, except the PTG factor spiritual change which was significantly correlated to positive reappraisal only (*r* = 0.48**; Table 4). Planful problem-solving and seeking social support coping strategies had the highest significant correlations with all PTG factors, except for spiritual change. Positive reappraisal showed a positive significant correlation with all the PTG factors; confrontive coping was also significantly correlated to all PTG factors except spiritual change (*r* = 0.003). The negative correlations were significant between the two coping strategies Distancing and Escape-avoidance and all the PTG factors, excluding spiritual change (*r* = −0.03) and (*r* = −0.01). Moreover, there was a positive significant correlation between self-controlling and three of the PTG factors: New possibilities (*r* = 0.42**), Personal Growth (*r* = 0.32*), and Appreciation of Life (*r* = 0.31*). As for accepting responsibility, there was no significant correlation between this coping strategy and all posttraumatic growth factors.

**Table 4:** Correlation between coping strategies and Posttraumatic growth factors (*N* = 60).

Coping strategies	Posttraumatic growth factors				
	Relating to	New possibilities	Personal growth	Spiritual change	Appreciation of life
Confrontive coping	0.40**	0.42**	0.40**	0.003	0.48**
Distancing	−0.53**	−0.40**	−0.30*	−0.03	−0.31*
Self-controlling	0.08	0.42**	0.32*	0.04	0.31*
Seeking social support	0.67**	0.61**	0.45**	0.17	0.54**
Accepting	−0.16	−0.07	0.007	0.12	−0.13
Escape-avoidance	−0.45**	−0.26*	−0.12	−0.01	−0.29*
Planful problem	0.64**	0.65**	0.50**	0.17	0.60**
Positive reappraisal	0.48**	0.53**	0.43**	0.48**	0.49**

*p < 0.05; **p < 0.01.

### Predictors of posttraumatic growth

A standard linear regression using a stepwise method was performed to identify which of the coping strategies were predictors of Posttraumatic Growth of adolescent refugees in the Center, Shatila. The total PTG score was regressed against various coping strategies to identify the variables that accounted for the largest variance in PTG. The results of this study showed that Planful Problem-solving and Seeking Social Support were the two predictors of posttraumatic growth among Palestinian adolescent refugees in Shatila Camp. Planful Problem-solving explained the highest variation 48.5% of the posttraumatic factors (*β* = 1.66/*p* < 0.01). Seeking Social Support added 12% of the variation in the posttraumatic factors (*β* = 1.96/*p* < 0.01). Table 5 reports the results of the regression.

**Table 5:** β coefficient of regression of posttraumatic growth factors among Palestinian refugees, (*N* = 60).

Coping strategies	*β*	Value of *p*	95% CI
Confrontive coping	0.14	0.16	–
Distancing	−0.18	0.23	–
Self-controlling	0.03	0.75	–
Seeking social support	1.96	<0.01*	[0.99, 2.92]
Accepting responsibility	−0.02	0.83	–
Escape-avoidance	*β* = −0.06	0.55	–
Planful problem solving	*β* = 1.66	<0.01*	[0.92, 2.40]
Positive reappraisal	*β* = 0.18	0.12	–

*p < 0.01.

On the other hand, the coping strategies Positive Reappraisal, Distancing, Confrontive coping, Escape-avoidance, Self-controlling, and Accepting responsibility did not enter the regression and were excluded due to non-significance. Therefore, the results showed that Planful Problem solving, and Seeking Social Support coping strategies utilized by adolescent refugees in the Shatila camp are significant predictors of PTG factors.

## Discussion and conclusion

The results of the study indicated that all the adolescents who participated in the study have experienced life stressful events. Most of the adolescents have reported several life stressful events like accidents, the loss of someone, witnessing a fight, etc. However, the pandemic was the most frequently experienced stressful event among the Palestinian refugees, followed by fire or explosion, and sudden accidental deaths. The results of this study reflect the current situation in Lebanon. The pandemic has been a common stressor among the participants and may be among the population of Shatila, as they had to stay at home, and this could have affected their family income and their social relationships with others. This is consistent with Gavin’s findings (2021). He described the pandemic as a radical change in the lives of people all around the world. Moreover, the participants reported an explosion or fire as a common stressor. This is related to the explosion of Beirut Port on the 4th of August 2020. The explosion destroyed more than one area of Beirut and its surrounding. Shatila camp has been affected and certain areas were destroyed. The findings of the present study were consistent with those of Gavin’s findings (2021). Gavin has identified the pandemic and the Beirut port explosion as the two most recent traumatic events in Lebanon. The country has faced dramatic economic and societal strains; the explosion of the port has added to the severe political and social crisis in Lebanon for decades. After the explosion, many adults and children were found under rubble or passed away; in addition, contact with others was lost.

Results of the study showed that the most prevalent coping strategies among Palestinian adolescent refugees are planful problem-solving, positive reappraisal, self-controlling, and accepting responsibility. These strategies represent problem-focused coping. Therefore, the results of this study confirm that problem-focused coping strategies are the most utilized among refugees in the Shatila camp. Problem-focused coping is when an individual gets task-oriented. In other words, a person uses problem-focused coping when they pursue new information, take different measures and responsibilities to change circumstances that create stress, manage stressful events, and generate possible and alternative solutions to a problem (e.g., reappraising the situation positively, seeking problem-solving advice from others, planning for solutions; Lazarus and Folkman, 1984; as cited by Compas et al., 2001). Planful problem solving is when individuals tend to think differently of a situation causing them an imbalance of thoughts and emotions, so they can overcome and solve it. Positive reappraisal is the adaptive way individuals use to rethink stressful events and interpret them as beneficial and valuable. The use of these coping strategies results in better health outcomes and develops the person’s growth. Research has demonstrated that the ability to find benefit from adversity is associated with improved health outcomes (Garland et al., 2009).

Planful problem-solving and seeking social support coping strategies had the highest significant positive correlations with all PTG factors, except Spiritual change. The more use of these coping strategies, the greater experience of posttraumatic growth. Seeking social support and Planful problem-solving coping strategies utilized by adolescent refugees in the Shatila camp were the significant predictors of all posttraumatic factors. On the other hand, other coping strategies did not predict posttraumatic growth among Palestinian refugees in the Shatila camp. Admittedly, Acar et al. (2021) and others discovered that problem-focused coping is correlated with PTG because it can lead to the reconstruction of essential assumptions regarding one’s identity, interpersonal connections, and spiritual beliefs to alleviate distress. Acar et al. (2021) discovered that engaging in problem-solving activities, reflecting on the traumatic incident, and learning to handle discomfort efficiently following trauma exposure all led to a sense of post-traumatic growth. In a similar vein, Ersahin (2022) discovered that problem-focused coping methods enhanced the PTG.

### Recommendations and implications

Based on this study’s findings, it is recommended that further research be conducted among Palestinian refugees from different age groups addressing different coping strategies other than the ones used in this study. Reconducting a study at a later time, after the pandemic is over and when the Beirut blast effects have been dealt with might have resulted in other stressful events among the refugees that are more related to their situation in the camp; therefore, different coping strategies could have been used and reported and possibly different PTG factors.

The results of this study have certain implications for refugees’ mental health. Psychologists and counselors could design innovative programs that are oriented toward Palestinian refugees and aim to strengthen the use of coping strategies. When working with Palestinian refugees, knowledge of the types of coping skills that have worked for them such as seeking social support and planful problem solving may enable educators and counselors to help them identify possible forms of coping that other refugees utilize. It is recommended to have a coping strategies program implemented in Shatila camp at its different schools and centers on regular basis to strengthen the various ways of coping among refugees. Psychiatric treatments and counseling sessions need to take initiative in providing proper intervention to overcome psychological stressors and maintain refugees’ psychological well-being. On further notice, nongovernmental organizations (NGOs), human rights organizations, and the UNHCR could support the actions and provide experts who can intervene and help refugees use more coping strategies, overcome psychological stressors and stressful events, and maintain their psychological well-being.

### Future directions and limitations

One of the limitations of this study was the sample size of adolescent Palestinian refugees. However, this was beyond the control of the researcher due to the pandemic and the circumstances of the country. Although the number of adolescents was sixty, the results of this study could not be generalized to all Palestinian adolescent refugees in different camps in Lebanon and the region. The environment and the circumstances in the camps are almost the same in different areas of Shatila or any other Palestinian camp in Lebanon; however, some external factors might affect the results of this study if it is to be conducted in another campsite. Another limitation of this study was the restriction of the age group to participants aged between 15 and 17, limiting the generalizability of the results to such age group. Involving different age groups of adolescents could have resulted in different coping strategies; therefore, different PTG factors. In addition, the study mainly used correlational research with its possible limitation. Further research could, for instance, investigate the coping strategies utilized by Palestinian adolescent refugees in a different context: the other branch of the center in the North Lebanon camp. Such research could contribute to identifying coping strategies that are common among Palestinian refugees who reside in different camps in Lebanon. Moreover, it is recommended that future research be conducted on a larger sample with a wider age range using experimental research. In addition, the study could be extended to other populations; for example, exploring the prevalent coping strategies among Syrian refugees living in the same campsite. The findings of this study will serve to compare the ways of coping used by different refugees living within the same circumstances.

## Data availability statement

The data analyzed in this study are available on request, all requests should be directed to beainy.sara@gmail.com.

## Ethics statement

The studies involving human participants were reviewed and approved by Institution Research Board-American University of Beirut. Written informed consent to participate in this study was provided by the participants’ legal guardian/next of kin.

## Author contributions

SB: data collection, data analysis and interpretation, and drafting the article. KH: conception or design of the work and critical revision of the article. All authors contributed to the article and approved the submitted version.

## Conflict of interest

The authors declare that the research was conducted in the absence of any commercial or financial relationships that could be construed as a potential conflict of interest.

## Publisher’s note

All claims expressed in this article are solely those of the authors and do not necessarily represent those of their affiliated organizations, or those of the publisher, the editors and the reviewers. Any product that may be evaluated in this article, or claim that may be made by its manufacturer, is not guaranteed or endorsed by the publisher.
